# The effect of astaxanthin after varicocele surgery on antioxidant status and semen quality in infertile men: A triple‐blind randomized clinical trial

**DOI:** 10.1002/fsn3.4365

**Published:** 2024-08-19

**Authors:** Shimal Ayub Mohammed Salih, Masoome Jabarpour, Mohammad Ali Sedighi Gilani, Hesamoddin Sajadi, Mojtaba Saedi Marghmaleki, Maryam Shabani Nashtaei, Maryam Salem, Fardin Amidi

**Affiliations:** ^1^ Department of Anatomy, School of Medicine Tehran University of Medical Sciences Tehran Iran; ^2^ Department of Infertility, Shariati Hospital Tehran University of Medical Sciences Tehran Iran; ^3^ Department of Andrology, Reproductive Biomedicine Research Center Royan Institute for Reproductive Biomedicine, ACECR Tehran Iran

**Keywords:** antioxidant status, astaxanthin, pro‐inflammatory cytokines, semen parameters, varicocele, varicocelectomy

## Abstract

Varicocele (VC) is widely recognized as a prevalent etiological factor contributing to male infertility. It has been established that the generation of reactive oxygen species (ROS) plays a significant role in the progression and development of VC. Antioxidants may regulate ROS levels in these patients. Astaxanthin (ASX) is a carotenoid compound with notable antioxidant and anti‐inflammatory characteristics. The current study postulated that the administration of ASX following varicocelectomy (VCT) could potentially enhance antioxidant status and semen quality in these patients. A total of 40 infertile males with clinical VC and abnormal semen analyses were randomly assigned to take part in the current trial. For 3 months following surgery, the intervention group took ASX (6 mg/day) while the control group received a placebo. After intervention, semen parameters, _antioxidant_ status, _and_ pro‐inflammatory cytokines were compared between the two groups. Regarding semen parameters, antioxidant treatment led to a significant improvement in total and progressive motility in the treatment group (*p* < 0.05). Additionally, ASX led to a considerable increase in the expression levels of NRF2, Keap1, SOD2, SOD3, and BCL2, though the enhancement in the expression level of SOD3 was not statistically significant (*p* > .05). However, ASX significantly decreased the BAX expression level (*p* < .05). Even though the level of total antioxidant capacity (TAC) of seminal fluid (SF) increased significantly in the treatment group (*p* < .05), the level of total oxidative stress (TOS) in SF did not differ substantially between treatment and control groups (*p* > .05). Based on inflammatory factors in SF, ASX led to a considerable reduction in levels of TNF‐α, IL‐1β, and IL‐6 (*p* < .05). Our findings demonstrated that ASX treatment provides an important contribution to VCT outcomes by modulating antioxidant status and pro‐inflammatory cytokines. Our results indicated that ASX may be beneficial as an adjuvant therapy for infertile men following VCT.

## Graphical abstract

Our study found that ASX treatment contributes to varicocelectomy outcomes by modulating antioxidant status and proinflammatory cytokines. It may be beneficial as an adjuvant therapy for infertile men following varicocelectomy.

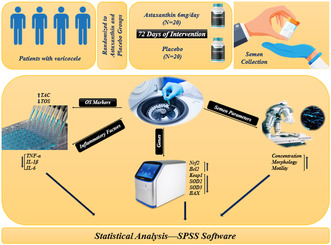

## 1 INTRODUCTION

Infertility is a medical condition that affects 15% of the population and is distinguished by the inability of individuals to conceive after at least one year of consistent and unprotected sexual activity. It is important to highlight that male‐related infertility constitutes nearly 50% of the documented cases, either as the primary cause or a contributing element (Hajian Monfared et al., 2017; Shokri et al., 2014). Several male causes of infertility have been identified (Ghorbani et al., 2014), but the precise etiology of infertility remains undetermined. The most prevalent correctable cause of male infertility is varicocele (VC), an aberrant dilation in the spermatic veins (Jensen et al., 2017). This particular defect accounts for approximately 35–50% of instances of primary infertility and up to 81% of cases of secondary infertility (Gorelick & Goldstein, 1993; Witt & Lipshultz, 1993). While there has been considerable research conducted on the pathophysiology of infertility caused by varicocele, the precise mechanisms underlying the adverse effects of VC on spermatogenesis remain unclear (Nematollahi‐Mahani et al., 2014). Nevertheless, recent research has elucidated that oxidative stress (OS) and a decrease in antioxidant levels—together with hyperthermia, hypoxia, hormonal imbalances, and inflammatory states—are significant contributors to the pathogenesis of VC (Hamada et al., 2013). OS, resulting from an excessive accumulation of reactive oxygen species (ROS), constitutes a significant factor in the etiology of infertility (Agarwal et al., 2014; Said et al., 2005). In recent years, there has been a well‐documented association and a direct correlation observed between levels of ROS in semen and the severity of VC (Hamada et al., 2013; Romeo et al., 2001; Smith et al., 2006). Several studies have provided evidence supporting the involvement of inflammatory pathways in the underlying mechanisms linked to the pathophysiology of infertility associated with VC (Hassanin et al., 2018). The SF of infertile men with VC may exhibit elevated levels of cytokines, including interleukins (IL‐1, IL‐6, IL‐37, and IL‐18) and tumor necrosis factor‐alpha (TNF‐α), due to OS induced by the heightened formation of ROS following hypoxia (Yilmaz et al., 2014). It is important to highlight that elevated levels of cytokines have been observed to correlate with decreased sperm motility, increased sperm necrosis, and aberrant spermatogenesis (Moretti et al., 2014). On the other hand, it is noteworthy that OS serves as an inducer for apoptosis, a process that is recognized as a contributing component to the pathogenesis of VC (Karna et al., 2019). Therefore, the implementation of various strategies to mitigate OS could potentially restore the fertility impaired by VC. Surgical intervention is often regarded as the gold‐standard therapeutic approach for the management of VC (Agarwal et al., 2007). Nevertheless, varicocelectomy (VCT) patients may not consistently exhibit the anticipated improvement in semen parameters; this has prompted the exploration of non‐invasive treatments such as antioxidant therapy (Kızılay & Altay, 2019). The potential advantages of combining antioxidants with VCT may surpass those of surgery performed alone (Finelli et al., 2022). The key rationale for the popularity of antioxidant therapy is that OS in VC is the principal mediator leading to testicular injury (Hamada et al., 2013). On the other hand, there is no evidence to suggest that surgery mitigates OS (Wang et al., 2019). By impeding the production of oxidative products, antioxidant therapy may aid in mitigating OS and ameliorating VC‐induced infertility. The findings of a systematic review and meta‐analysis suggest that antioxidant therapy following VCT may enhance sperm quality and create a more favorable environment for spermatozoa by reducing FSH levels (Wang et al., 2019).

Astaxanthin (ASX) is a xanthophyll carotenoid with a red‐orange hue that occurs naturally in several organisms, including algae, plants, and animal species such as crabs, salmon, shrimp, flamingos, and quail (Naguib, 2000). ASX has been shown in studies to have various clinical advantages and pharmacological effects, such as antioxidant, antitumor, anti‐cancer, anti‐diabetic, neuroprotective, and anti‐inflammatory characteristics (Kohandel et al., 2021). The antioxidant properties of ASX have been confirmed to occur through the activation of the nuclear factor erythroid 2‐related Factor 2 (NRF2) signaling pathway (Kohandel et al., 2021). NRF2, a key cellular redox homeostasis detector, plays a crucial role in maintaining cellular redox balance. The release of NRF2 from Keap1—an inhibitory protein—modulates the transcription of cytoprotective genes like NADPH quinone oxidoreductase‐1 (NQ‐1), heme oxygenase‐1 (HO‐1), and superoxide dismutase (SOD) (Baird & Yamamoto, 2020; Loboda et al., 2016). NRF2 is also a potent inhibitor of apoptosis, making its activation beneficial in various disorders linked to OS (Khan et al., 2018). Our laboratory found that ASX protects against OS in PCOS patients and in ex vivo human granulosa cells (HGCs) by activating the NRF2 signaling pathway (Eslami et al., 2021; Gharaei et al., 2022). Additionally, ASX ameliorates apoptosis and ER stress in PCOS patients, according to previous findings (Jabarpour et al., 2023, 2024). Numerous studies explore ASX's impact on OS indicators and the NRF2‐ARE pathway, but no clinical trials specifically investigate its effects on infertile men with varicocele. Consequently, the study aimed to investigate the influence of ASX on the expression of NRF2‐ARE pathway genes, semen parameters, and SF levels of OS and inflammatory markers in infertile men undergoing VCT.

## 2 METHODS

### 2.1 Trial design

This study was a prospective, 1:1 ratio, parallel, triple‐blind, randomized clinical trial (RCT). A total of 40 patients diagnosed with VC II/III and experiencing infertility were included in this study. These patients had sub‐inguinal VCT at the Royan Institute in Tehran, Iran, throughout the period from December 2021 to May 2023. The VC was classified in accordance with World Health Organization (WHO) criteria (Rowe et al., 2000) and confirmed using Doppler ultrasonography. The assessment of deviations in sperm parameters, such as sperm count, morphology, and motility, was conducted using two distinct semen analyses. Patients diagnosed with VC and exhibiting abnormal sperm parameters were scheduled for VCT. Male patients aged 18–40 years, 18.5 < BMI < 30, and with infertility history ≥12 months were included in the study. The study excluded individuals who met the following criteria: those who had previously undergone surgery related to the genitourinary system and/or VC, those with idiopathic infertility, azoospermia, severe oligospermia, Sertoli cell‐only syndrome, and leukocytospermia, those who had a medical condition affecting fertility and had received treatment for fertility within the past 3 months, those with a history of undescended testis, testicular cancer, testicular trauma, post‐pubertal mumps, endocrine disorder, or obstructive urogenital disease, those who followed a diet specifically aimed at improving fertility, those who consumed excessive amounts of alcohol, cigarettes, drugs, opioids, or hallucinogens, those with a positive HIV serology, or those who had an acute infection and another identifiable cause of infertility. The project was granted approval by the Ethics Committee of Tehran University of Medical Sciences (IR.TUMS.MEDICINE.REC.1401.354) in strict adherence to the pertinent guidelines and regulations. Information on the trial was uploaded to the website of the Iranian Registry of Clinical Trials (IRCT) (IRCT‐ID: IRCT20230924059501N1). The informed consent form was endorsed by every participant prior to the intervention.

### 2.2 Randomization and blinding

**FIGURE 1:**
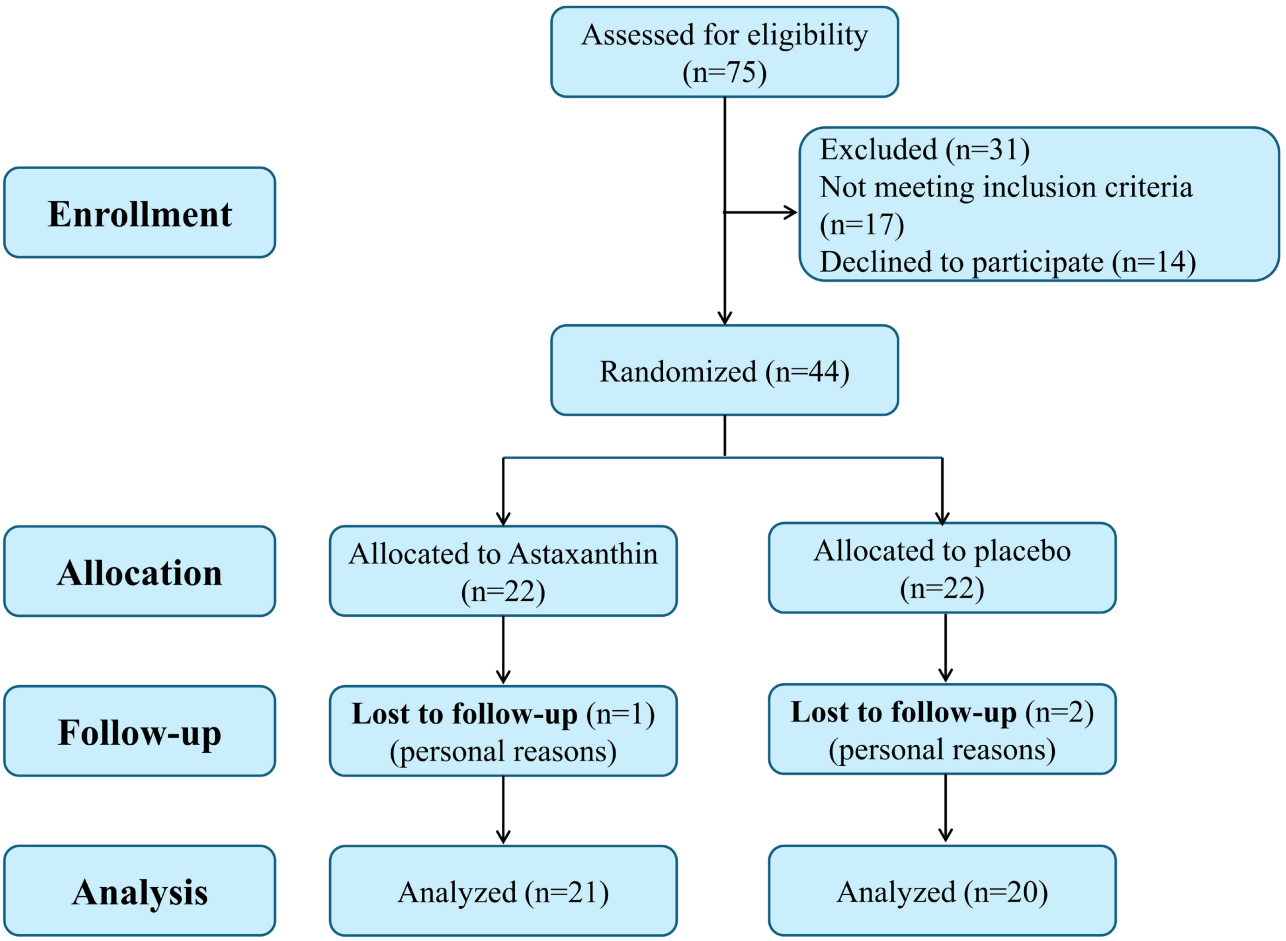
Summary of patient flow through the study.

Participants were randomly assigned to the control (placebo) and intervention (ASX) groups via blocked randomization following VCT. The blocks had a dimension of four and were comprised of the letters A and B, which stood for the intervention and control groups, respectively. The contents were concealed within opaque containers that were sequentially numbered and sealed. During randomization, participants were kept separate from other researchers to prevent bias. Figure 1 depicts the map of the investigation. The decipherer was not a member of the research team, and the statistician, patients, and investigator were all oblivious to the grouping in this study. The trial participants were allocated ASX and placebo capsules at random from bottles containing 90 capsules each. Notably, the medicinal contents of each container were encoded by an individual external to the research team, and the research team was not privy to the code's interpretation.

### 2.3 Intervention

The participants in the therapy group were orally administered ASX 6 mg/day (Astareal, Tokyo, Japan) for 90 days. In the placebo group, the patients were administered a daily dose of 1 capsule containing edible paraffin for 90 days. The appearance of the placebo capsule was identical to that of ASX. A comparable company manufactured the capsules at the same time. The optimum dose of ASX is 6 mg administered orally daily, as indicated by a prior study (Brendler & Williamson, 2019). In addition to adhering to their customary daily regimen, participants were required to inform the researchers of any alterations to their dietary or physical activity practices. To guarantee compliance throughout the experiment, all participants were provided with daily prompts to consume their supplements and were also requested to return the empty supplement containers.

### 2.4 Surgical procedure

The sub‐inguinal VCT procedure was carried out in all patients utilizing optical magnification (HEINE Cx2.3 Binocular Loop, Dusseldorf, Germany) to protect the arteries and lymphatic system. The procedure was performed under either general or regional anesthesia while the patients were lying on their backs. In each instance, an incision measuring roughly 2–3 cm was performed. The spermatic cord was raised and positioned on a penrose drain. The enlarged veins were tied off while keeping the artery and lymphatic vessels intact. During the postoperative follow‐up period, semen analysis was repeated. All VCTs were conducted by the same specialist.

### 2.5 Semen analysis

Patients' sperm were obtained through masturbation following 3–4 days of sexual abstinence. Sperm motility, morphology, and count were evaluated at two time points: prior to the VCT and 3 months subsequent to the VCT, following the intake of ASX or placebo. Sperm samples were collected in polypropylene containers. Within half an hour after sample collection, the samples were liquefied and analyzed in accordance with the World Health Organization (WHO) Manual for Semen Analysis (2010) (WHO, 2010). All laboratory analyses were conducted by experts who were not privy to the study protocol.

### 2.6 Seminal fluid separation and biochemical measurement

Followed by routine semen analysis, the remaining semen samples were centrifuged at 12,000 ***g*** for 5 min. The upper layer of SF was collected and stored in aliquots at −80°C for the determination of biochemical markers. SF was obtained from all patients before and after the intervention to evaluate OS markers (TAC and TOS activities) and pro‐inflammatory cytokines (IL‐1β, IL‐6, and TNF‐α). To minimize inter‐assay variations and systematic error, OS markers and cytokines were quantified in duplicate, blinded, and within the same analytical run. The levels of OS markers in SF were quantified in accordance with the manufacturer's guidelines using human enzyme‐linked immunosorbent assay (ELISA) kits (Zellbio, GmbH, Germany). Utilizing a microplate reader (BioTek, Winooski, VT, USA), absorbance at wavelengths unique to each marker was determined. Concentrations of pro‐inflammatory cytokines in SF were ascertained using human ELISA kits (KPG, Iran; Karmania Pars Gene Company). The procedure adhered to the guidelines provided by the manufacturer. The absorbance was assessed by a microplate reader (BioTek, Winooski, VT, USA).

### 2.7 RNA extraction and real‐time polymerase chain reaction

In short, total RNA was manually extracted from sperm cells through a solution of RNX‐PLUS (SinaClon, Tehran, Iran) based on the manufacturer's protocol. The quantification of RNA was performed by assessing the absorbance ratio A260/A280 (using the Biochrom WPA Biowave, Cambridge, UK). Next, a commercial reverse transcription kit (SinaClon, Tehran, Iran) was used to generate complementary DNA (cDNA). The RealQ Plus 2 × Master Mix Green (Amplicon, Denmark) was used to determine the levels of gene expression. In addition, PCRs were performed using particular primers for NRF2, Keap1, SOD2, SOD3, BAX, BCL2, and the glyceraldehyde‐3‐phosphate dehydrogenase (GAPDH) as a reference gene. The reactions were conducted in duplicate using a Light Cycler 96 System (Roche, Germany). Data analysis was performed using the 2^−ΔΔCT^ technique. The primers were designed using the software Beacon Designer 7, and their details can be observed in Table 1.

**TABLE 1:** Specific primers used for real‐time quantitative PCR.

		Primer	Annealing *tm*	Melting *tm*	Product size (bp)
GAPDH	Forward Reverse	GTGGTCTCCTCTGACTTCAAC GGAAATGAGGCTTGACAAAGTGG	59	84.8	97
NRF2	Forward Reverse	CGACGGAAAGAGTATGAGCTGG GCATCTGATTTGGGAATGTGGG	60	82.4	215
KEAP1	Forward Reverse	AGACGTGGACTTTCGTAGCC CCAGGAACGTGTGACCATCA	60	85.9	111
BAX	Forward Reverse	TTTCTGACGGCAACTTCAACTG TCCAATGTCCAGCCCATGA	59	85.5	127
Bcl2	Forward Reverse	CATCACAGGACTTCTGCGAATAC CCCATCAATCTTCAGCACTCTCC	60	86.8	245
SOD2	Forward Reverse	GTGAACAACCTGAACGTCACC CCCTTTGGGTTCTCCACCAC	60	84.2	171
SOD3	Forward Reverse	CTTGGAGGAGCTGGAAAGGTG CCTTGGCGTACATGTCTCGG	60	85.3	158

Abbreviations: BAX, Bcl‐2‐associated X protein; Bcl2, B‐cell lymphoma 2; GAPDH, glyceraldehyde‐3‐Phosphate dehydrogenase; KEAP1, Kelch‐like ECH‐associated protein 1; NRF2, nuclear factor erythroid 2‐related Factor 2; SOD2, Superoxide dismutase 2; SOD3, Superoxide dismutase3.

### 2.8 Statistical analysis

The data were analyzed using GraphPad Prism 9. The mean ± standard deviation (SD) was used to describe numeric variables. The data's distribution was assessed using the Kolmogorov–Smirnov test. Comparisons were conducted using either a paired or an unpaired Student's *t*‐test, with a significance level of *p* < .05. Recent studies (Rostami et al., 2023) revealed that the difference between the two treatment effects in TNF‐α is 0.448, and the polled standard deviation of both comparison groups is 0.5365. So, considering the 80% power and also the 5% type 1 error, based on the statistical superiority design, 18 people in each group were included in this trial. After accounting for a 20% loss to follow‐up, the sample size was determined to be 22 patients in each group.

## 3 RESULTS

**FIGURE 2:**
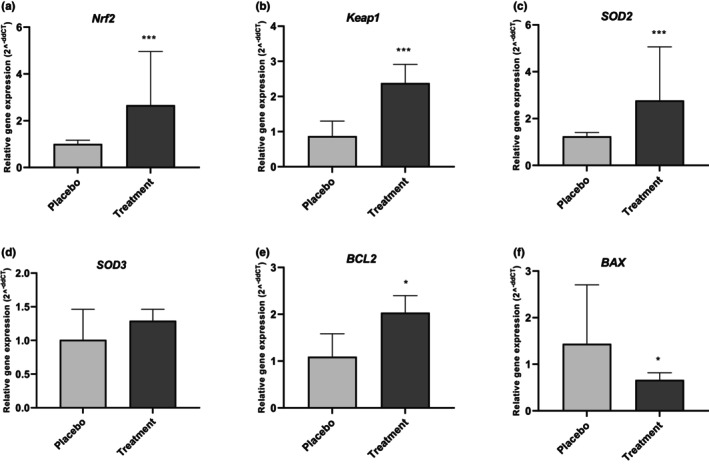
The fold changes levels of Nrf2 (a), Keap1 (b), SOD2 (c), SOD3 (d), BCL2 (e), and BAX (f) in sperm cells of the placebo and treatment groups. Statistical significance (*p* < .05) was assessed by a *t*‐test. The results showed that the fold change level of BAX was significantly decreased in the intervention group (*p* < .05). After intervention, it was found that in the ASX group, the fold change levels of Nrf2, Keap1, SOD2, SOD3, and BCL2 were significantly increased compared to the control group, while the induction level of SOD3 was not significant between the two groups (*p* > .05). Differences between groups: *p < .05, ***p < .001.

Generally, during the intervention stage, three subjects had to withdraw from the study, including two subjects in the placebo group and one case in the ASX group (all for personal reasons). A total of 41 patients were involved in this study, with 21 patients in the intervention group and 20 patients in the placebo group (Figure 1). The participants in the trial did not report any negative symptoms or effects associated with the ASX supplementation. Additionally, they demonstrated strong adherence to the intervention. At the start of the trial, there were no notable disparities in the average age, BMI, hormonal profile, VC grade and laterality, testis volume, and sperm parameters between the treatment and control groups (Table 2). According to gene expression results, the treatment group exhibited significantly higher expression levels of NRF2 (*p* = .007; Figure 2a), Keap1 (*p* = .004; Figure 2b), SOD2 (*p* = .008; Figure 2c), and BCL2 (*p* = .016; Figure 2e). While the mRNA expression level of SOD3 was increased in the treatment group (*p* = .168; Figure 2d), this increase did not reach statistical significance. In contrast, the level of BAX expression decreased substantially (*p* = .044; Figure 2f) compared to the placebo group. The findings of the SF analysis (Table 3) indicate that the TAC level in the treatment group increased significantly (*p* < .05). However, the TOS level of SF did not differ significantly between the treatment and control groups (*p* > .05). Also, Table 3 indicated that the decrease in SF levels of TNF‐α, IL‐1β, and IL‐6 were statistically significant in the treatment group compared to the placebo group. Sperm parameters are presented in Table 4. There was no noticeable difference in the sperm parameters in the placebo group after treatment when comparing the baseline values. However, after ASX therapy, the treatment group showed a noticeable improvement in count, total count, and total motility compared to the baseline values. Based on Table 4, there was statistically significant improvement in total motility (*p* = .048) and progressive motility (*p* = .006) in the treatment group after intervention compared to the placebo group. Although other sperm parameters also improved after intervention, this improvement was not significant.

**TABLE 2:** Baseline parameters in individual groups.

	Placebo (*n* = 20)	Treatment (*n* = 21)	*p*‐value
Age (Year)	36.90 ± 4.76	35.80 ± 4.61	.463
BMI (kg/m^2^)	24.16 ± 1.84	24.41 ± 1.60	.649
FSH (mIU/mL)	5.35 ± 1.91	4.67 ± 1.33	.204
Testosterone (ng/mL)	4.62 ± 1.67	4.54 ± 1.53	.871
Infertility (%)			
Primary	8 (40%)	5 (24%)	.326
Secondary	12 (60%)	16 (76%)	
Varicocele grade (%)			
Grade II	7 (35%)	10 (47%)	.716
Grade III	13 (65%)	11 (55%)	
Varicocele laterality (%)			
Unilateral	16 (80%)	18 (86%)	.696
Bilateral	4 (20%)	3 (14%)	
Testis volume (mL)			
Right testis	19.11 ± 1.65	18.49 ± 2.64	.383
Left testis	19.12 ± 1.57	18.33 ± 2.67	.259
Sperm parameters			
Volume (CC)	3.94 ± 1.00	4.16 ± 1.44	.576
Count (×10^6^/mL)	46.10 ± 41.63	41.16 ± 38.72	.574
Total count (×10^6^)	182.6 ± 171.7	176 ± 183.7	.545
Total motility (%)	41.40 ± 20.09	46.58 ± 20.46	.232
Progressive motility (%)	11.50 ± 11.66	16.47 ± 12.58	.208
Morphology (%)	1.30 ± 1.17	1.50 ± 1.35	.621

*Note*: Data based on mean ± SD (independent *t*‐test) or number (%) (^c^ *χ* ^2^ test).

Abbreviations: BMI, body mass index; FSH, follicle‐stimulating hormone.

**TABLE 3:** Comparison of SF levels of OS markers and pro‐inflammatory cytokines after treatment.

	Placebo (*n* = 20)	Treatment (*n* = 21)	*p*‐value
IL1β (pg/mL)	12.67 ± 1.16	8.57 ± 0.71	<.0001***
IL‐6 (pg/mL)	13.93 ± 2.87	11.67 ± 1.0	<.0001***
TNFα (pg/mL)	20.07 ± 1.8	15.70 ± 1.17	.002**
TOS (μmol/L)			
Sperm lysates	1.87 ± 0.25	2.16 ± 0.69	.091
Seminal plasma	5.13 ± 1.0	4.09 ± 1.53	.194
TAC (mmol/L)			
Sperm lysates	0.343 ± 0.01	0.363 ± 0.02	.001**
Seminal plasma	3.18 ± 1.29	3.59 ± 0.52	.016*

*Note*: Data based on mean ± SD (independent *t*‐test).

Abbreviations: IL1β, interlucin‐1β; IL‐6, interlucin‐6; OS, oxidative stress; SF, semen fluid; TNF‐α, tumor necrosis factor‐α; TOS, total oxidative status; TAC, total antioxidant capacity.

Statistically significant *p < .05, **p < .01 and ***p < .001.

**TABLE 4:** Comparison of semen parameters between two groups.

Parameters		Placebo (*n* = 20)	Treatment (*n* = 21)	*p* ^1^
Volume (CC)	Before	3.94 ± 1.00	4.16 ± 1.44	.576
	After	4.20 ± 1.26	4.08 ± 1.53	.720
	Change	0.30 ± 0.94	0.12 ± 1.41	.648
	*p* ^2^	.170	.741	
Count (×10^6^/mL)	Before	46.10 ± 41.63	41.16 ± 38.72	.574
	After	54.93 ± 37.41	56.42 ± 41.04	.809
	Change	3.75 ± 6.54	4.75 ± 5.34	.599
	*p* ^2^	.109	.0008***	
Total count (×10^6^)	Before	182.6 ± 171.7	176 ± 183.7	.545
	After	225.5 ± 154.3	241.9 ± 227.1	.591
	Change	42.89 ± 140.40	74.74 ± 194.90	.556
	*p* ^2^	.062	.001**	
Total motility (%)	Before	41.40 ± 20.09	46.58 ± 20.46	.232
	After	59.10 ± 21.31	58.75 ± 23.57	.023*
	Change	4.55 ± 7.48	9.55 ± 8.01	.048*
	*p* ^2^	.121	.001**	
Progressive motility (%)	Before	11.50 ± 11.66	16.47 ± 12.58	.208
	After	17.15 ± 13.34	19.39 ± 10.74	.575
	Change	3.80 ± 8.18	10.50 ± 6.44	.006**
	*p* ^2^	.054	.086	
Morphology (%)	Before	1.30 ± 1.17	1.50 ± 1.35	.621
	After	1.70 ± 1.30	1.95 ± 1.84	.623
	Change	0.40 ± 0.68	0.45 ± 1.35	.251
	*p* ^2^	.096	.154	

*Note*: Data based on mean ± SD, *p* ^1^ (independent *t*‐test), *p* ^2^ (paired *t*‐test). Statistically significant *p < .05, **p < .01 and ***p < .001.

## 4 DISCUSSION

Our research revealed that antioxidant treatment had a beneficial impact on sperm parameters by modulating antioxidant status and pro‐inflammatory cytokines after VCT. Surgical intervention is often regarded as the gold‐standard therapeutic approach for the management of VC (Agarwal et al., 2007). Nevertheless, VCT patients may not consistently exhibit the anticipated improvement in semen parameters; this has prompted the exploration of non‐invasive treatments such as antioxidant therapy (Kızılay & Altay, 2019). The results of the current clinical trial examining the impact of ASX on infertile patients following VCT indicated that ASX decreased the SF level of TOS but increased the TAC level. A 90‐day course of ASX also increased the mRNA expression levels of BCL2, NRF2, Keap1, SOD2, and SOD3 in the current study. In contrast, BAX mRNA expression was diminished compared to the placebo group. According to our findings, ASX significantly decreased the concentrations of TNF‐α, IL‐1β, and IL‐6 in SF. In VC, OS serves as the primary mediator of testicular injury. ROS production is stimulated by exposure to heat, hypoxia, and toxic adrenal and renal metabolites. ROS are critical regulators of physiological processes, including capacitation, acrosome reactions, hyperactivation, and fertilization. However, antioxidant systems are responsible for regulating their concentration within physiological ranges. An excess of ROS can result in detrimental consequences such as DNA damage, protein oxidation, and lipid peroxidation (Baskaran et al., 2021). A correlation has been observed between elevated levels of oxidation–reduction potential (ORP), which serves as an indicator of redox balance in seminal plasma, and diminished semen quality in patients with VC (Agarwal et al., 2016). Comparing VC patients to healthy controls, a meta‐analysis found that VC patients had elevated ROS levels and decreased TAC (Agarwal et al., 2006). As one of the most potent antioxidants known (Sztretye et al., 2019), ASX has been reported to exert a protective effect against OS and can effectively improve OS in humans and animals (Baralic et al., 2013; Choi et al., 2011; Park et al., 2010; Pereira et al., 2021; Sztretye et al., 2019). ASX may also enhance the translocation of NRF2 to the nucleus, which increases the expression of endogenous antioxidants (Mahmoud et al., 2004). The present study finds a decrease in TOS level in the treatment group, but the decrease was not significant. However, ASX increased the TAC level of SF significantly. Our findings were consistent with those of prior research. Demir et al. showed that ASX protected testicular tissue against damage caused by torsion/detorsion by exerting antioxidant effects and suppressing endoplasmic stress. The findings demonstrated that ASX treatments led to a reduction in TOS and OSI levels and an increase in TAC levels in the testicular tissue (Demir et al., 2022). Similarly, it has been observed that ASX has a protective effect on intestinal tissue by reducing the levels of ROS and OSI while increasing the levels of TAC (Akduman et al., 2021). Our prior research validated that ASX supplementation for 60 days increased the follicular fluid (FF) level of TAC, whereas the FF levels of malondialdehyde (MDA) and SOD did not differ significantly between the treatment and control groups (Jabarpour et al., 2023). Based on multiple animal experiments, it has been observed that ASX has the ability to decrease OS by reducing the levels of 8‐hydroxy‐2′‐deoxyguanosine (8‐OHdG) (Kochi et al., 2014) and MDA (Guo et al., 2015). However, it can also increase the activity of antioxidant enzymes (Fassett & Coombes, 2011; Guo et al., 2015). Only a few clinical trials have been conducted in this direction. A systematic review and meta‐analysis of ASX's antioxidant effect in humans found that ASX may reduce OS by decreasing specific lipid peroxidation (ISP and MDA) and improving plasma antioxidant capability (TAC) and a specific antioxidant enzyme (SOD). However, ASX's antioxidant properties in humans are unknown (Wu et al., 2020).

It has been established that VC is a chronic inflammatory vascular disease that can result in a chronic inflammatory response at the local level. OS induces a chronic inflammatory response within the body system and damages biomolecules. Several investigations have verified that proteins associated with inflammatory pathways have distinct expression patterns in individuals with VC. Patients with VC have shown increased levels of pro‐inflammatory cytokines in their SF (Zeinali et al., 2017). VC promotes the secretion of pro‐inflammatory and inflammatory cytokines, including IL‐1β, IL‐6, IL‐8, and TNF‐α (Habibi et al., 2015). Extensive in vivo and in vitro investigations have demonstrated that ASX exhibits anti‐inflammatory properties in mammals. This finding provides encouragement for the potential of ASX as a therapeutic intervention for inflammation‐related disorders (Chang & Xiong, 2020). The anti‐inflammatory effects of ASX treatment are demonstrated through significant inhibition of inflammatory mediators' activity, including inducible nitric oxide synthase (iNOS), cyclooxygenase‐2 (COX‐2), and matrix metalloproteinases (MMPs) expression (Talukdar et al., 2020). A substantial body of in vitro and in vivo research has demonstrated that ASX inhibits the production of pro‐inflammatory cytokines, including IL‐1β, IL‐6, and TNF‐α (Campoio et al., 2011). ASX has been demonstrated to decrease TNF‐α, IL‐1β, IL‐6, and IL‐18 by regulating MAPK/NF‐kB and JAK/STAT (Cai et al., 2019; Fu et al., 2020; Park et al., 2010b; Talukdar et al., 2020b). Recently, we indicated that ASX significantly decreased serum levels of IL‐1β, IL‐6, and TNF‐α in endometriosis patients (Rostami et al., 2023). Furthermore, ASX therapy might mitigate acute lung injury induced by cecal ligation puncture by inhibiting the release of pro‐inflammatory cytokines caused by OS (Bi et al., 2017). In the present study, ASX significantly decreased the levels of TNF‐α, IL‐1β, and IL‐6 in SF. This result is in accordance with previous research.

Research has focused on understanding how ASX regulates pro‐inflammatory cytokines through various signaling pathways. The NF‐B pathway is a key component. Additionally, ASX regulates the NRF2 and sirtuin1 pathways, which are crucial for its anti‐inflammatory activity (Talukdar et al., 2020).

NRF2, as a transcription factor, enhances the expression of antioxidant proteins and protects against oxidative injury (Ngo & Duennwald, 2022). The antioxidant properties of ASX are mediated by NRF2 signaling, which has beneficial effects on various disorders associated with OS (Cheng & Eroglu, 2021; Kanwugu & Glukhareva, 2023). NRF2 is also an effective inhibitor of apoptosis, as evidenced by numerous studies (Khan et al., 2018; Liu et al., 2017; Niture & Jaiswal, 2012). NRF2 is recognized to be linked to testicular apoptosis triggered by diabetes (Jiang et al., 2014). Through apoptosis, VC induces testicular impairment as one of its primary mechanisms (Wang et al., 2018). The findings from the study conducted by Yong Wang revealed that VC causes oxidative and mitochondrial apoptotic damage. However, the administration of GSPE (grape seed proanthocyanidin extract) increases the expression of NRF2 and its downstream genes, which helps to alleviate this damage and restore normal function of the spermatogenic organs (Wang et al., 2018). Multiple research studies have provided evidence that ASX exerts its antioxidative function by activating the NRF2 pathway (Ashrafizadeh et al., 2022; Kohandel et al., 2021b). However, there is limited research that has specifically examined the relationship between ASX and the NRF2 signaling pathway in the testis. According to Wang & Zhuang (2019) ASX therapy has the ability to restore the NRF2/HO‐1 signaling pathway in sperm cells at both the mRNA and protein levels following exposure to LPS. Our prior research demonstrated that ASX ameliorated the OS in HGCs (Eslami et al., 2021) and PCOS patients (Gharaei et al., 2022) by increasing the expression of HO‐1 and NQ‐1 through NRF2 activation. ASX was able to substantially increase the expression of NRF2, thereby enabling it to regulate the expression of downstream preserver genes, as demonstrated by the results of our previous research, which are consistent with the findings of the current study. In the current investigation, ASX administration increased the levels of the preservative genes SOD2 and SOD3. Keap1 is a principal inhibitor of NRF2 activity. This molecule inhibits the nuclear translocation of NRF2 by forming a complex with it (Baird & Yamamoto, 2020). As a result, we anticipated that an increase in NRF2 expression would lead to a decrease in Keap1 expression. However, our real‐time analysis showed an increase in the expression of Keap1, which contrasts with the results of our experimental study on the effects of ASX on HGCs (Eslami et al., 2021). This phenomenon may be attributed to the negative feedback mechanism that exists between NRF2 and Keap1. According to Lu et al., positive feedback exists between NRF2 and Keap1, so an increase in NRF2 expression will result in an increase in Keap1 expression. Alternatively stated, their cellular abundance is governed by an autoregulatory loop involving Keap1 and NRF2 (Lee et al., 2007). Aside from the NRF2 cytosolic binding caused by the inhibitory action of Keap1, there are other factors that can also control the transcription of the NRF2 gene. Recent investigations have emphasized several different pathways, such as microRNAs (Cheng et al., 2013), protein kinases phosphorylating NRF2 (Bryan et al., 2013; Cuadrado, 2015), and acetylation of NRF2 (Sun et al., 2009). Hence, the observed augmentation in Keap1 expression could potentially be a biological reaction to the heightened NRF2 expression resulting from alternative regulatory mechanisms. It is imperative to consider the disparity between mRNA expression and protein levels. Consequently, the authors cannot exclude the possibility that these variations in Keap1 protein levels and mRNA expression exist in these patients. Additional research may elucidate the precise cause of this observation. The accumulation of evidence demonstrates that there is a direct connection between the therapeutic effects of ASX and its anti‐apoptotic properties (Fakhri et al., 2019). The findings of the current investigation demonstrated increased expression of BCL2 and decreased expression of BAX in the treatment group in comparison to the control group. The findings from our prior investigation align with the results of the current study, which showed that ASX has the ability to enhance BCL2 expression and reduce BAX expression in the GCs of PCOS patients (Jabarpour et al., 2024). Based on the beneficial function of NRF2 activation in numerous diseases commonly associated with OS and the inhibitory effect of NRF2 on apoptosis, it can be hypothesized that ASX inhibits apoptosis via NRF2 activation.

Finally, analysis of our results showed that all sperm parameters improved in the treatment group. However, there was a statistically significant change in both total and progressive motility. Although the use of antioxidants for sub‐fertile patients has been suggested in a number of studies, their application following VCT has been rarely mentioned in the literature. Our findings are consistent with recent research that examined the effects of several antioxidants on semen quality following VCT. Ardestani Zadeh et al. (2019), in their work, assessed the effects of Vit E‐Se‐FA antioxidants on sperm parameters following VCT. Their findings demonstrated that both the count and motility of sperm showed improvement 6 months after receiving supplements subsequent to VCT. In a similar study, Azizollahi et al. (2013) examined how zinc sulfate, FA, and zinc/FA affected protamine concentration, acrosomal integrity, and sperm quality after VCT. They provided evidence that FA administration increased sperm count. Zinc sulfate (ZS) enhanced the morphology of the sperm. The ZS and FA groups exhibited a substantial increase in protamine content and halo formation rate (Azizollahi et al., 2013). Lu et al. (2018) found in a placebo‐controlled trial that supplementing with melatonin, a potent antioxidant, 3 months following VCT resulted in additional advantages in terms of enhanced sperm parameters, total antioxidant capacity, and hormonal profile. A recent meta‐analysis demonstrated that the use of antioxidant treatment following VCT can enhance fertility results by primarily enhancing sperm concentration, motility, DNA integrity, and serum follicle‐stimulating hormone levels (Chen et al., 2018). Similarly, a separate meta‐analysis examining the effectiveness of antioxidant therapy on assessments of sperm quality following VCT demonstrated notable enhancements in sperm concentration, motility, progressive motility, and morphology in the antioxidant group 3 months after VCT. In relation to the outcomes seen after a period of 6 months, there was an improvement in sperm parameters, except sperm motility and progressive sperm motility (Wang et al., 2019). Curiously, another recent study demonstrated that different antioxidants had no impact on the quality of semen in a larger cohort of infertile individuals (Steiner et al., 2018). Several more trials (Bozhedomov et al., 2017; Rafiee et al., 2016; Raigani et al., 2014; Safarinejad et al., 2011) also yielded similar findings, indicating that antioxidants had no discernible impact. These studies suggest that antioxidants are intriguing, but the clinical data should be interpreted with caution.

According to our knowledge, two clinical trials have been carried out to examine the impact of ASX on male fertility. The initial experiment included individuals with a prior medical history of male infertility, irrespective of semen characteristics. The findings demonstrated positive alterations in the concentration of inhibin B, linear velocity of sperm, levels of reactive ROS, and pregnancy rate (Comhaire et al., 2005). The second RCT assessed the effectiveness of oral intake of ASX on semen parameters in patients with oligoasthenoteratozoospermia (OAT). Nevertheless, no statistically significant alterations were detected. The inadequate initial values for basic sperm parameters observed in the second study indicate that ASX might not yield favorable results when semen quality is exceptionally poor (Kumalic et al., 2020).

The fact that all of the VCTs were carried out by the same specialist provides a significant advantage for our research. However, this research also has several limitations. Due to the limited size of our sample, it may be a challenge to identify subtle variations in response to ASX therapy. Furthermore, the duration of our study's follow‐up period was limited; while some outcomes did not show significant changes, they may hold significance when observed over a longer length of time. The research was constrained by the absence of an objective assessment of patient adherence; specifically, the serum or plasma concentrations of ASX were not quantifiable in this investigation. Moreover, it is vital to evaluate the discrepancy between mRNA expression and protein levels. Therefore, it may have been more advantageous if the authors had assessed the protein levels of the genes.

Numerous studies on antioxidant treatments in male infertility have contradictory results, making it difficult to compare agents and make definitive judgments. However, our study found that ASX treatment contributes to VCT outcomes by modulating antioxidant status and pro‐inflammatory cytokines. It may be beneficial as adjuvant therapy for infertile men following VCT, but further studies with larger sample sizes are needed to make a proper decision on ASX supplementation post‐VCT.

## AUTHOR CONTRIBUTIONS

**Shimal Ayub Mohammed Salih:** Data curation (lead); investigation (lead); project administration (lead); validation (equal); visualization (equal); writing – review and editing (supporting). **Masoome Jabarpour:** Formal analysis (lead); methodology (supporting); writing – original draft (lead). **Mohammad Ali Sedighi Gilani:** Data curation (lead); investigation (supporting); project administration (equal); validation (equal); visualization (equal); writing – review and editing (equal). **Hesamoddin Sajadi:** Investigation (supporting); project administration (equal); visualization (equal); writing – review and editing (equal). **Mojtaba Saedi Marghmaleki:** Formal analysis (supporting); investigation (supporting); software (equal); validation (equal); writing – review and editing (equal). **Maryam Shabani Nashtaei:** Methodology (supporting); validation (equal); visualization (equal); writing – review and editing (equal). **Maryam Salem:** Investigation (supporting); project administration (supporting); writing – review and editing (equal). **Fardin Amidi:** Conceptualization (lead); investigation (equal); resources (lead); supervision (lead); validation (equal); visualization (equal); writing – review and editing (equal).

## FUNDING INFORMATION

This study was financially supported by the Tehran University of Medical Sciences, Tehran, Iran.

## CONFLICT OF INTEREST STATEMENT

The authors declare no conflicts of interest.

## ETHICS STATEMENT

The Ethics Committee of Tehran University of Medical Sciences approved the project (Ethics Committee reference number: IR.TUMS.REC.1399.340).

## CONSENT TO PARTICIPATE

Written consent was obtained from all participants.

## Data Availability

The datasets used and analyzed during the present study are available from the corresponding author on reasonable request.
